# Supplementary material for: Fetal abdominal obesity in women with one value abnormality on diagnostic test for gestational diabetes mellitus
Source: PLoS One. 2024 Jun 4;19(6):e0304875. doi: 10.1371/journal.pone.0304875 (PMC11149842; doi:10.1371/journal.pone.0304875)
Supplement: S1 File — (DOCX) [file pone.0304875.s001.docx]

**S1 Figure. Flow chart of study population**


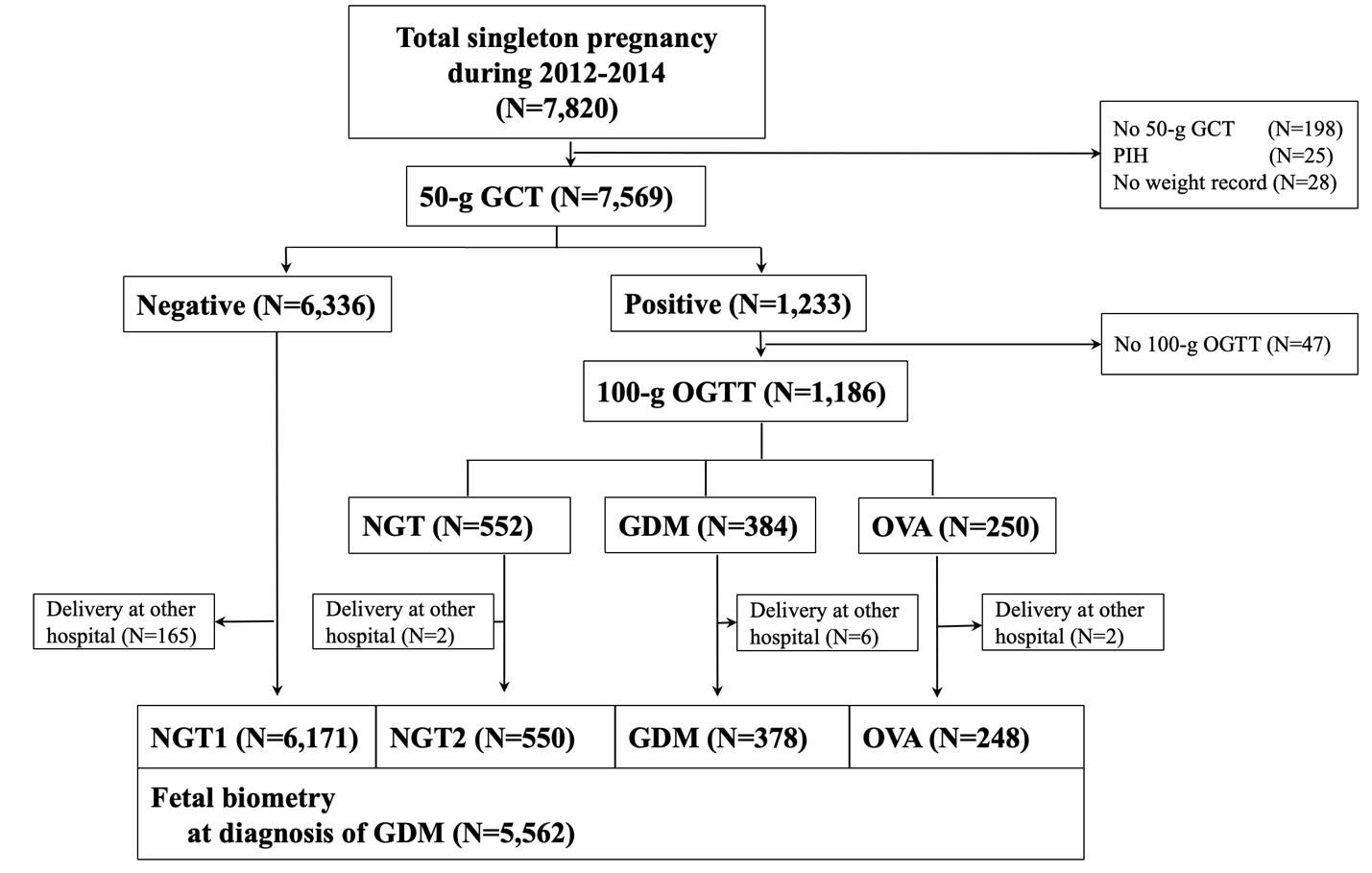


**S1 Table. Prevalence of OVA according to maternal age and pre-pregnancy BMI**

|  | Age <35 years  (n=4,927) | Age ≥35 years  (n=2,642) | Total |
| --- | --- | --- | --- |
| BMI <25 kg/m^2^  (n=7,079) | Group 1  2.5%  (117/4665) | Group 3  4.1% ^a^  (99/2414) | 3.0%  (214/7079) |
| BMI ≥ 25 kg/m^2^  (n=490) | Group 2  6.5%^a^  (17/262) | Group 4  7.5%^a,b^  (17/228) | 6.9%^d^  (34/490) |
| Total  (n=7,569) | 2.7%  (132/4927) | 4.4%^c^  (116/2642) | 3.3%  (250/7569) |

OVA, one value abnormality; BMI, body mass index.

^a^p <0.05 compared to Group 1; ^b^p <0.05 compared to Group 3; ^c^p <0.05 compared to Age <35 years; ^d^p <0.05 compared to BMI <25 kg/m^2^

**S2 Table. Prevalence of FAO and pregnancy outcome in the subjects with OVA according to maternal age and pre-pregnancy BMI**

|  | **NGT1**  (n=4686) | **OVA** | | | |
| --- | --- | --- | --- | --- | --- |
|  |  | Group 1  (n=79) | Group 2  (n=14) | Group 3  (n=70) | Group 4  (n=11) |
| FAO (%) | 9.5 | 10.1 | 7.1 | 12.9 | 27.3^a^ |
| LGA (%) | 5.4 | 7.6 | 21.4 ^a^ | 8.6 | 18.2 |
| Macrosomia (%) | 2.1 | 3.8 | 14.3 ^a^ | 4.3 | 0 |
| Primary C-sec (%) | 21.2 | 24.1 | 35.7 | 37.1 ^a^ | 27.2 |

FAO, fetal abdominal obesity; OVA, one value abnormality; BMI, body mass index; NGT, normal glucose tolerance; LGA, large for gestational age, Group 1 (age <35 years and BMI <25 kg/m^2^), Group 2 (age <35 years and BMI ≥25 kg/m^2^), Group 3 (age ≥35 years and BMI <25 kg/m^2^), and Group 4 (age ≥35 years and BMI ≥25 kg/m^2^).

^a^p <0.05 compared to NGT1

**S3 Table. Clinical characteristics and pregnancy outcomes according to the time point showing abnormal glucose value on 3-hr 100-g OGTT in the OVA subjects.**

|  | OVA-0  (n=13) | OVA-1  (n=40) | OVA-2  (n=75) | OVA-3  (n=46) | p-value |
| --- | --- | --- | --- | --- | --- |
| Clinical |  |  |  |  |  |
| Age (years) | 35.9±3.2 | 34.8±4.2 | 34.5±3.3 | 33.5±3.9 | 0.1450 |
| Pre-pregnancy BMI (kg/m^2^) | 24.9±3.8 | 21.8±3.7^a^ | 21.0±2.8 ^a^ | 21.9±3.7 ^a^ | 0.0026 |
| Weight gain (kg), |  |  |  |  |  |
| Pre-pregnancy ̶ at diagnosis | 7.8±2.6 | 8.3±2.9 | 7.0±2.8 | 8.0±3.4 | 0.1175 |
| HbA1c at diagnosis (%) | 5.3±0.2 | 5.1±0.3 | 5.1±0.3 | 5.2±0.3 | 0.0527 |
| HOMA-IR | 3.1±0.9 | 2.1±1.1 | 1.8±0.9 ^a^ | 2.17±0.97 | 0.0246 |
| HOMA-ß | 128.9±40.2 | 155.0±82.0 | 156.5±87.14 | 188.0±61.2 | 0.3109 |
| FAO (+) at diagnosis (%) | 30.8 | 5.0 | 13.3 | 10.9 | 0.0961 |
| **Pregnancy outcomes** |  |  |  |  |  |
| Primipara (%) | 61.5 | 67.5 | 66.7 | 56.5 | 0.6622 |
| Male sex of infant (%) | 61.54 | 52.5 | 50.7 | 60.8 | 0.6774 |
| LGA (%) | 7.7 | 16.2 | 11.4 | 13.3 | 0.8439 |
| Macrosomia (%) | - | 5.4 | 7.1 | 4.4 | 0.7467 |
| Primary cesarean delivery(%) | 46.2 | 25.0 | 33.3 | 21.7 | 0.2618 |

OGTT, oral glucose tolerance test; OVA, one value abnormality; BMI, body mass index; HOMA-IR, homeostatic model assessment for insulin resistance; HOMA-β, homeostatic model assessment for insulin secretion; FAO, fetal abdominal obesity; LGA, large for gestational age.

^a^p< 0.05, compared with OVA-0, - OVA-0 (fasting), OVA-1 (1hr after glucose load), OVA-2 (2hr after glucose load), and OVA-3 (3hr after glucose load)
