# Supplementary material for: Fetal abdominal obesity in women with one value abnormality on diagnostic test for gestational diabetes mellitus
Source: PLoS One. 2024 Jun 4;19(6):e0304875. doi: 10.1371/journal.pone.0304875 (PMC11149842; doi:10.1371/journal.pone.0304875)
Supplement: S1 Dataset — (ZIP) [file pone.0304875.s002.zip › 14 PLOS One_analysis code.docx]

// last edited: 04/16/2024

// author: Soo Kyung Park, University of Texas Health Science Center at Houston

// note: This code shares the steps of data analysis done using statistical software STATA 15.1 for the manuscript

// "Fetal Abdominal Obesity in Women with One Value Abnormality on Diagnostic Test for Gestational Diabetes Mellitus"

// contents: I. IMPORT DATA

// II. MAIN TEXT RESULTS -> TABLE 1 - TABLE 5

***********************************************;

** I. IMPORT DATA

***********************************************;

** import the data ;

import excel "C:\Users\skpar\Documents\gdm\PLOS One_dataset.xlsx", sheet("Sheet1") firstrow

***************************************************************************************;

** II. TABLE 1. Clinical and biochemical characteristics in NGT, OVA, and GDM subjects

***************************************************************************************;

****************************;

** clinical characteristics ;

****************************;

** descriptive statistics ;

** total NGT ;

sum age Ht PPTwt PGwt Termwt PPTwt_BMI PGwt_BMI Termwt_BMI weight_change weight_change2 if ngt_gdm_total_binary == 0

** NGT1 vs. NGT2 vs. OVA vs. GDM ;

bysort dx_rere: sum age Ht PPTwt PGwt Termwt PPTwt_BMI PGwt_BMI Termwt_BMI weight_change weight_change2

** pairwise comparison ;

** age ;

oneway age dx_rere

pwmean age, over(dx_rere) mcompare(tukey) effects

** height ;

oneway Ht dx_rere

pwmean Ht, over(dx_rere) mcompare(tukey) effects

** pre-pregnancy weight ;

oneway PPTwt dx_rere

dunntest PPTwt, by(dx_rere)

kwallis PPTwt, by(dx_rere)

** at-diagnosis weight ;

oneway PGwt dx_rere

dunntest PGwt, by(dx_rere)

kwallis PGwt, by(dx_rere)

** near-term weight ;

oneway Termwt dx_rere

dunntest Termwt, by(dx_rere)

kwallis Termwt, by(dx_rere)

** pre-pregnancy BMI ;

oneway PPTwt_BMI dx_rere

dunntest PPTwt_BMI, by(dx_rere)

kwallis PPTwt_BMI, by(dx_rere)

** at-diagnosis BMI ;

oneway PGwt_BMI dx_rere

dunntest PGwt_BMI, by(dx_rere)

kwallis PGwt_BMI, by(dx_rere)

** near-term BMI ;

oneway Termwt_BMI dx_rere

dunntest Termwt_BMI, by(dx_rere)

kwallis Termwt_BMI, by(dx_rere)

** weight gain - Prepregnancy ~ at diagnosis ;

oneway weight_change dx_rere

dunntest weight_change, by(dx_rere)

kwallis weight_change, by(dx_rere)

** weight gain - At diagnosis ~ near term ;

oneway weight_change2 dx_rere

pwmean weight_change2, over(dx_rere) mcompare(tukey) effects

*******************************;

** biochemical characteristics ;

*******************************;

** descriptive statistics ;

** total NGT ;

sum PG1 glucose0 glucose00 glucose60 glucose600 glucose120 glucose1200 glucose180 glucose1800 ///

HbA1C Ins0 Ins1 homa_ir homa_beta if ngt_gdm_total_binary == 0

** NGT1 vs. NGT2 vs. OVA vs. GDM ;

bysort dx_rere: sum PG1 glucose0 glucose00 glucose60 glucose600 glucose120 glucose1200 glucose180 glucose1800 ///

HbA1C Ins0 Ins1 homa_ir homa_beta

** pairwise comparison ;

** 50g-GCT ;

oneway PG1 dx_rere

dunntest PG1, by(dx_rere)

** 100g-OGTT - fasting ;

oneway glucose0 dx_rere

dunntest glucose0, by(dx_rere)

** 100g-OGTT - 1hr ;

oneway glucose60 dx_rere

dunntest glucose60, by(dx_rere)

** 100g-OGTT - 2hr ;

oneway glucose120 dx_rere

dunntest glucose120, by(dx_rere)

** 100g-OGTT - 3hr ;

oneway glucose180 dx_rere

dunntest glucose180, by(dx_rere)

** HbA1c ;

oneway HbA1C dx_rere

dunntest HbA1C, by(dx_rere)

** insulin - fasting ;

oneway Ins0 dx_rere

pwmean Ins0, over(dx_rere) mcompare(tukey) effects

** insulin - 1hr ;

oneway Ins1 dx_rere

dunntest Ins1, by(dx_rere)

** HOMA-IR ;

oneway homa_ir dx_rere

dunntest homa_ir, by(dx_rere)

** HOMA-BETA ;

oneway homa_beta dx_rere

pwmean homa_beta, over(dx_rere) mcompare(tukey) effects

*******************************************************************************************************************;

** II. TABLE 2. Results of fetal biometry and FAORs measured at diagnosis, 24-28 GW in NGT, OVA, and GDM subjects

*******************************************************************************************************************;

** fetal biometry ;

** descriptive statistics ;

** total NGT ;

sum PGwk AC BPD FL EST if ngt_gdm_total_binary == 0 & AC_PGwk != .

** NGT1 vs. NGT2 vs. OVA vs. GDM ;

bysort dx_rere : sum PGwk AC BPD FL EST

** pairwise comparison ;

** GA-LMP ;

oneway PGwk dx_rere

dunntest PGwk, by(dx_rere)

** GA-AC ;

oneway AC dx_rere

pwmean AC, over(dx_rere) mcompare(tukey) effects

** GA-BPD ;

oneway BPD dx_rere

pwmean BPD, over(dx_rere) mcompare(tukey) effects

** GA-FL ;

oneway FL dx_rere

dunntest FL, by(dx_rere)

** EFW ;

oneway EST dx_rere

pwmean EST, over(dx_rere) mcompare(tukey) effects

** FAOR ;

** descriptive statistics ;

** total NGT ;

sum AC_PGwk AC_BPD AC_FL if ngt_gdm_total_binary == 0 & AC_PGwk != .

** NGT1 vs. NGT2 vs. OVA vs. GDM ;

bysort dx_rere : sum AC_PGwk AC_BPD AC_FL

** pairwise comparison ;

** GA-AC/GA-LMP ;

oneway AC_PGwk dx_rere

pwmean AC_PGwk, over(dx_rere) mcompare(tukey) effects

oneway AC_PGwk ngt_ova_binary

ttest AC_PGwk, by(ngt_ova_binary)

** GA-AC/GA-BPD ;

oneway AC_BPD dx_rere

pwmean AC_BPD, over(dx_rere) mcompare(tukey) effects

oneway AC_BPD ngt_ova_binary

ttest AC_BPD, by(ngt_ova_binary)

** GA-AC/GA-FL ;

oneway AC_FL dx_rere

dunntest AC_FL, by(dx_rere)

oneway AC_FL ngt_ova_binary

ttest AC_FL, by(ngt_ova_binary)

*****************************************************************************************;

** II. TABLE 3. Odds ratio for FAO at diagnosis, 24-28 GW in NGT2, OVA, and GDM subjects

*****************************************************************************************;

logistic AC_PGwk_YN i.dx_rere , nolog

logistic AC_BPD_YN i.dx_rere , nolog

logistic AC_FL_YN i.dx_rere , nolog

** GA-AC/GA-LMP ≥ 90th percentile ;

logistic AC_PGwk_YN i.dx_01_binary, nolog

logistic AC_PGwk_YN i.dx_02_binary, nolog

logistic AC_PGwk_YN i.dx_03_binary, nolog

** GA-AC/GA-BPD ≥ 90th percentile ;

logistic AC_BPD_YN i.dx_01_binary, nolog

logistic AC_BPD_YN i.dx_02_binary, nolog

logistic AC_BPD_YN i.dx_03_binary, nolog

** GA-AC/GA-FL ≥ 90th percentile ;

logistic AC_FL_YN i.dx_01_binary, nolog

logistic AC_FL_YN i.dx_02_binary, nolog

logistic AC_FL_YN i.dx_03_binary, nolog

***************************************************************************************************************;

** II. TABLE 4. Prevalence of FAO at diagnosis, 24-28 GW, and pregnancy outcomes in NGT, OVA, and GDM subjects

***************************************************************************************************************;

** FAO at diagnosis ;

tab2 AC_PGwk_YN dx_rere , chi col row

prtest AC_PGwk_YN , by(ngt1_ngt2_binary)

prtest AC_PGwk_YN , by(ngt1_ova_binary)

prtest AC_PGwk_YN , by(ngt1_gdm_binary)

prtest AC_PGwk_YN , by(ngt2_ova_binary)

prtest AC_PGwk_YN , by(ngt2_gdm_binary)

prtest AC_PGwk_YN , by(ova_gdm_binary)

** Primipara ;

tab2 primipara_binary_reverse dx_rere , chi col row

prtest primipara_binary_reverse , by(ngt1_ngt2_binary)

prtest primipara_binary_reverse , by(ngt1_ova_binary)

prtest primipara_binary_reverse , by(ngt1_gdm_binary)

prtest primipara_binary_reverse , by(ngt2_ova_binary)

prtest primipara_binary_reverse , by(ngt2_gdm_binary)

prtest primipara_binary_reverse , by(ova_gdm_binary)

** LGA ;

tab2 lga_binary dx_rere , chi col row

prtest lga_binary , by(ngt1_ngt2_binary)

prtest lga_binary , by(ngt1_ova_binary)

prtest lga_binary , by(ngt1_gdm_binary)

prtest lga_binary , by(ngt2_ova_binary)

prtest lga_binary , by(ngt2_gdm_binary)

prtest lga_binary , by(ova_gdm_binary)

** Macrosomia ;

tab2 macro_binary dx_rere , chi col row

prtest macro_binary , by(ngt1_ngt2_binary)

prtest macro_binary , by(ngt1_ova_binary)

prtest macro_binary , by(ngt1_gdm_binary)

prtest macro_binary , by(ngt2_ova_binary)

prtest macro_binary , by(ngt2_gdm_binary)

prtest macro_binary , by(ova_gdm_binary)

** Infant birth weight ;

oneway Wt dx_rere

dunntest Wt, by(dx_rere)

kwallis Wt, by(dx_rere)

** Gestational age at delivery ;

bysort dx_rere : sum IUP

oneway IUP dx_rere

dunntest IUP , by(dx_rere)

kwallis IUP , by(dx_rere)

** Male sex of infant ;

tab2 sex_binary_reverse dx_rere , chi col row

prtest sex_binary_reverse , by(ngt1_ngt2_binary)

prtest sex_binary_reverse , by(ngt1_ova_binary)

prtest sex_binary_reverse , by(ngt1_gdm_binary)

prtest sex_binary_reverse , by(ngt2_ova_binary)

prtest sex_binary_reverse , by(ngt2_gdm_binary)

prtest sex_binary_reverse , by(ova_gdm_binary)

** Primary cesarean delivery ;

tab2 primary_c_sec dx_rere , chi col row

prtest primary_c_sec , by(ngt1_ngt2_binary)

prtest primary_c_sec , by(ngt1_ova_binary)

prtest primary_c_sec , by(ngt1_gdm_binary)

prtest primary_c_sec , by(ngt2_ova_binary)

prtest primary_c_sec , by(ngt2_gdm_binary)

prtest primary_c_sec , by(ova_gdm_binary)

** Comparison of clinical characteristics by NGT vs. GDM among OVA subjects

tab ngt_ova_binary , m

tab ngt_ova_binary AC0_Prewk_YN , m

ttest age if ngt_ova_binary == 1 , by(AC0_Prewk_YN)

ttest PPTwt_BMI if ngt_ova_binary == 1 , by(AC0_Prewk_YN)

ttest weight_change if ngt_ova_binary == 1 , by(AC0_Prewk_YN)

ttest HbA1C if ngt_ova_binary == 1 , by(AC0_Prewk_YN)

ttest homa_ir if ngt_ova_binary == 1 , by(AC0_Prewk_YN)

ttest homa_beta if ngt_ova_binary == 1 , by(AC0_Prewk_YN)

ttest glucose0 if ngt_ova_binary == 1 , by(AC0_Prewk_YN)

ttest glucose00 if ngt_ova_binary == 1 , by(AC0_Prewk_YN)

tab AC_PGwk_YN AC0_Prewk_YN if ngt_ova_binary == 1, chi col row

prtest AC_PGwk_YN if ngt_ova_binary == 1, by(AC0_Prewk_YN)

tab primipara_binary_reverse AC0_Prewk_YN if ngt_ova_binary == 1, chi col row

prtest primipara_binary_reverse if ngt_ova_binary == 1, by(AC0_Prewk_YN)

tab sex_binary AC0_Prewk_YN if ngt_ova_binary == 1, chi col row

prtest sex_binary if ngt_ova_binary == 1, by(AC0_Prewk_YN)

tab lga_binary AC0_Prewk_YN if ngt_ova_binary == 1, chi col row

prtest lga_binary if ngt_ova_binary == 1, by(AC0_Prewk_YN)

tab macro_binary AC0_Prewk_YN if ngt_ova_binary == 1, chi col row

prtest macro_binary if ngt_ova_binary == 1, by(AC0_Prewk_YN)

tab mod_binary AC0_Prewk_YN if ngt_ova_binary == 1, chi col row

prtest mod_binary if ngt_ova_binary == 1, by(AC0_Prewk_YN)

tab primary_c_sec AC0_Prewk_YN if ngt_ova_binary == 1, chi col row

prtest primary_c_sec if ngt_ova_binary == 1, by(AC0_Prewk_YN)

********************************************************************************************;

** II. TABLE 5. Clinical characteristics and pregnancy outcomes by the presence or absence

** of FAO at 24-28 GW in OVA subjects

********************************************************************************************;

** clinical ;

** Age ;

ttest age if dx_re == 2 , by(AC_PGwk_YN)

** Pre-pregnancy BMI ;

ttest PPTwt_BMI if dx_re == 2 , by(AC_PGwk_YN)

** Weight gain - Pre-pregnancy ~ at diagnosis ;

ttest weight_change if dx_re == 2 , by(AC_PGwk_YN)

ttest weight_change if dx_re == 2 , by(AC0_Prewk_YN)

** HbA1c at diagnosis ;

ttest HbA1C if dx_re == 2 , by(AC_PGwk_YN)

** HOMA-IR ;

ttest homa_ir if dx_re == 2 , by(AC_PGwk_YN)

ttest homa_ir if ngt_ova_binary == 1 , by(AC0_Prewk_YN)

** HOMA-BETA ;

ttest homa_beta if dx_re == 2 , by(AC_PGwk_YN

ttest homa_beta if ngt_ova_binary == 1 , by(AC0_Prewk_YN)

bysort AC_PGwk_YN : sum homa_ir homa_beta if ngt_ova_binary ==1 , d

ttest homa_ir if dx_re == 2 , by(AC0_Prewk_YN)

ttest homa_beta if dx_re == 2 , by(AC0_Prewk_YN)

** pregnancy outcomes ;

** Primipara ;

tab2 primipara_binary_reverse AC_PGwk_YN if dx_re == 2 , chi col row

tab primipara_binary_reverse AC_PGwk_YN if ngt_ova_binary == 1, chi col row

prtest primipara_binary_reverse if ngt_ova_binary == 1, by(AC_PGwk_YN)

** Male sex of infant ;

tab2 sex_binary AC_PGwk_YN if dx_re == 2 , chi col row

tab sex_binary AC_PGwk_YN if ngt_ova_binary == 1, chi col row

prtest sex_binary if ngt_ova_binary == 1, by(AC_PGwk_YN)

** LGA ;

tab2 lga_binary AC_PGwk_YN if dx_re == 2 , chi col row

tab lga_binary AC_PGwk_YN if ngt_ova_binary == 1, chi col row

prtest lga_binary if ngt_ova_binary == 1, by(AC_PGwk_YN)

** Macrosomia ;

tab2 macro_binary AC_PGwk_YN if dx_re == 2 , chi col row

tab macro_binary AC_PGwk_YN if ngt_ova_binary == 1, chi col row

prtest macro_binary if ngt_ova_binary == 1, by(AC_PGwk_YN)

** Cesarean delivery ;

tab2 mod_binary AC_PGwk_YN if dx_re == 2 , chi col row

tab mod_binary AC_PGwk_YN if ngt_ova_binary == 1, chi col row

prtest mod_binary if ngt_ova_binary == 1, by(AC_PGwk_YN)

** Primary cesarean delivery ;

tab2 primary_c_sec AC_PGwk_YN if dx_re == 2 , chi col row

tab primary_c_sec AC_PGwk_YN if ngt_ova_binary == 1, chi col row

prtest primary_c_sec if ngt_ova_binary == 1, by(AC_PGwk_YN)
